# Population modifiable risk factors associated with neonatal mortality in 35 sub-Saharan Africa countries: analysis of data from demographic and health surveys

**DOI:** 10.1016/j.eclinm.2024.102682

**Published:** 2024-06-20

**Authors:** Kedir Y. Ahmed, Subash Thapa, Tahir A. Hassen, Teketo Kassaw Tegegne, Abel F. Dadi, Daniel Bogale Odo, Habtamu Mellie Bizuayehu, Desalegn Markos Shifti, Sewunet Admasu Belachew, Getiye Dejenu Kibret, Daniel Bekele Ketema, Zemenu Yohannes Kassa, Erkihun Amsalu, Meless G. Bore, Abdulbasit Seid, Yonatan M. Mesfin, Kelemu Tilahun Kibret, M. Mamun Huda, Shakeel Mahmood, Anayochukwu E. Anyasodor, Allen G. Ross

**Affiliations:** a Rural Health Research Institute, Charles Sturt University, Orange, NSW 2800, Australia; b Center for Women’s Health Research, College of Health, Medicine and Wellbeing, The University of Newcastle, NSW, Australia; c Institute for Physical Activity and Nutrition, Deakin University, Geelong, VIC, Australia; d Menzies School of Health Research, Charles Darwin University, Australia; e Addis Continental Institute of Public Health, Addis Ababa, Ethiopia; f National Centre for Aboriginal and Torres Strait Islander Wellbeing Research, National Centre for Epidemiology and Population Health, Australian National University, Canberra, ACT, Australia; g First Nations Cancer and Wellbeing (FNCW) Research Program, School of Public Health, The University of Queensland, Qld, Australia; h Child Health Research Centre, The University of Queensland, Qld, Australia; i Faculty of Medicine, Health and Human Sciences, Macquarie University, Australia; j The George Institute for Global Health, University of New South Wales (UNSW), Sydney, Australia; k School of Public Health, College of Medicine and Health Science, Debre Markos University, Ethiopia; l College of Medicine and Health Sciences, Hawassa University, Hawassa, Ethiopia; m School of Nursing and Midwifery, University of Technology Sydney, Sydney, Australia; n Sydney Medical School, Faculty of Medicine and Health, University of Sydney, Australia; o St. Paul Hospital Millennium Medical College, Addis Ababa, Ethiopia; p School of Nursing, College of Medicine and Health Science, Hawassa University, Hawassa, Ethiopia; q Australian Living Evidence Collaborations, School of Public Health and Prevention Medicine, Monash University, Australia; r SAEFVIC, Infection and Immunity, Murdoch Children’s Research Institute, 50 Flemington Road, Parkville, VIC, Australia; s Global Centre for Preventive Health and Nutrition, Institute for Health Transformation, School of Health and Social Development, Faculty of Health, Deakin University, Geelong, VIC, Australia

**Keywords:** Neonatal mortality, Population attributable fractions, Sub-Saharan Africa, Sustainable development goals (SDGs)

## Abstract

**Background:**

Sub-Saharan Africa (SSA) has the highest burden of neonatal mortality in the world. Identifying the most critical modifiable risk factors is imperative for reducing neonatal mortality rates. This study is the first to calculate population-attributable fractions (PAFs) for modifiable risk factors of neonatal mortality in SSA.

**Methods:**

We analysed the most recent Demographic and Health Surveys data sets from 35 SSA countries conducted between 2010 and 2022. Generalized linear latent and mixed models were used to estimate odds ratios (ORs) along with 95% confidence intervals (CIs). PAFs adjusted for communality were calculated using ORs and prevalence estimates for key modifiable risk factors. Subregional analyses were conducted to examine variations in modifiable risk factors for neonatal mortality across Central, Eastern, Southern, and Western SSA regions.

**Findings:**

In this study, we included 255,891 live births in the five years before the survey. The highest PAFs of neonatal mortality among singleton children were attributed to delayed initiation of breastfeeding (>1 h after birth: PAF = 23.88%; 95% CI: 15.91, 24.86), uncleaned cooking fuel (PAF = 5.27%; 95% CI: 1.41, 8.73), mother’s lacking formal education (PAF = 4.34%; 95% CI: 1.15, 6.31), mother’s lacking tetanus vaccination (PAF = 3.54%; 95% CI: 1.55, 4.92), and infrequent antenatal care (ANC) visits (PAF = 2.45; 95% CI: 0.76, 3.63). Together, these five modifiable risk factors were associated with 39.49% (95% CI: 21.13, 48.44) of neonatal deaths among singleton children in SSA. Our subregional analyses revealed some variations in modifiable risk factors for neonatal mortality. Notably, delayed initiation of breastfeeding consistently contributed to the highest PAFs of neonatal mortality across all four regions of SSA: Central, Eastern, Southern, and Western SSA.

**Interpretation:**

The PAF estimates in the present study indicate that a considerable proportion of neonatal deaths in SSA are preventable. We identified five modifiable risk factors that accounted for approximately 40% of neonatal deaths in SSA. The findings have policy implications.

**Funding:**

None.

**Research in context**

**Evidence before this study**

Sub-Saharan Africa (SSA) has the highest burden of neonatal mortality globally. This study aimed to identify population-modifiable risk factors linked to neonatal mortality in SSA. We searched PubMed, from database inception to October 20, 2023, for published papers without language restrictions, using the terms “neonatal mortality,” “neonatal deaths,” “newborn mortality,” and “newborn deaths,” along with “population attributable fractions,” “population attributable risk,” and “modifiable risk factors” specific to SSA. Our search yielded 269 results. However, these studies predominantly relied on relative measures of associations such as odds ratios (ORs) or relative risks (RRs), which might not provide direct estimates for effective public health planning and resource allocation. To enhance the efficiency of policymakers and public health professionals in SSA, it is crucial to provide estimates of population-attributable fractions (PAFs) for modifiable risk factors of neonatal mortality.

**Added value of this study**

This study used the most recent nationally representative Demographic and Health Survey (DHS) data sets to estimate the PAFs of modifiable risk factors for neonatal mortality in 35 SSA countries. PAFs adjusted for communality were calculated using ORs and prevalence estimates for key modifiable risk factors. Our findings showed that delayed initiation of breastfeeding, use of uncleaned cooking fuel, mothers lacking formal education, mothers lacking tetanus vaccination, and infrequent antenatal care visits were attributed to 39.5% of neonatal deaths among singleton children in SSA.

**Implications of all the available evidence**

The PAF estimates in the present study indicate that a considerable proportion of neonatal deaths in SSA are preventable. It is recommended to allocate resources towards promoting breastfeeding, tackling household air pollution, improving maternal education, and enhancing the overall perinatal continuum of care.

## Introduction

Improving child survival is a global health priority. The first 28 days of life, also known as the neonatal period, is the most critical period for ensuring a child's survival.^1^ In 2021, five million children under the age of five years lost their lives worldwide, with 2.3 million of these deaths occurring within the first month of life.^1^ Given this fact, the world nations endorsed the Sustainable Development Goals (SDGs) Target 3.2 aiming to reduce the neonatal mortality rate to 12 per 1000 live births by 2030.^2^ However, without additional investments in healthcare, 63 nations globally will fall short of this neonatal mortality goal by 2030, and many of these countries are in sub-Saharan Africa (SSA).^1^

Sub-Saharan Africa has the highest burden of neonatal mortality globally, with 27 deaths per 1000 live births, contributing to 45% of global newborn deaths.^1^ A child born in SSA is 10 times more likely to die in the first month than a child born in a high-income country.^1^ United Nations (UN) multi-agency estimates showed that 43 out of 48 countries in the SSA are projected to miss the SDG neonatal mortality target by 2030, and approximately half of them may only meet the SDG target by 2050 if current progress does not accelerate.^1^ Previous studies from the SSA countries reported various risk factors associated with increased neonatal mortality. These include suboptimal breastfeeding,^3^ lack of postnatal health visits,^4^ low birth weight,^5^ lack of maternal tetanus vaccination during pregnancy,^6^ inadequate antenatal care (ANC) visits,^3^^,^^7^ poor socioeconomic households,^8^ and lack of maternal education.^9^

While previous studies in SSA have identified risk factors linked to neonatal mortality, these studies primarily used relative measures of association such as odds ratios (ORs) and relative risks (RRs).^5^^,^^10^ However, relying on these relative measures of association may not be the most effective strategy for public health planning and resource allocation.^11^^,^^12^ The public health importance of an association between a factor and an outcome might be limited when the factor is rare, but it could have a more substantial impact if the factor is common. Policymakers and public health professionals in SSA can benefit from population-attributable fraction (PAF) estimates for modifiable risk factors.^13^

Unlike the relative measures of associations (e.g., RRs and ORs), PAF provides the estimate for understanding both the strength of the relationship between the risk factor and disease and the prevalence of the risk factor in the population.^12^, ^13^, ^14^ This enables an estimate of the proportion of a specific outcome that can be potentially attributed to an exposure in the study population, as well as the potential reduction in the outcome if the exposure prevalence were hypothetically reduced to zero. It is important to acknowledge key assumptions of PAF, including the independence of exposures and the unidirectional and constant associations between exposures and outcomes over time.^15^^,^^16^ Despite its limitations, PAF estimates offer a concise method of quantifying risk and can complement other approaches in identifying modifiable risk factors for policy intervention and prioritisation.

To effectively address neonatal mortality through targeted interventions, a critical first step involves the identification of key modifiable risk factors. This study used nationally representative Demographic and Health Survey (DHS) data sets to examine the modifiable risk factors associated with neonatal mortality in 35 SSA countries. The study findings will be instrumental in reducing the burden of neonatal deaths in SSA and achieving the SDGs by 2030.^2^

## Methods

### Study design and data sources

We analysed population-based data sets from the most recent DHS conducted in 35 SSA countries between 2010 and 2022. These countries were grouped into four World Health Organization (WHO) regions: Central SSA (Angola, Congo Brazzaville, Congo Democratic Republic, and Gabon), Eastern SSA (Burundi, Comoros, Ethiopia, Kenya, Madagascar, Malawi, Mozambique, Rwanda, Tanzania, Uganda, and Zambia), Southern SSA (Lesotho, Namibia, South Africa, and Zimbabwe), and Western SSA (Benin, Burkina Faso, Cameroon, Chad, Côte d'Ivoire, Gambia, Ghana, Guinea, Liberia, Mali, Mauritania, Niger, Nigeria, Senegal, Sierra Leone, and Togo). Appendix 1 P 1 shows the sample size and response rate for each respective country survey.

The DHS surveys are conducted by the health ministry or governmental agencies of each respective country, with support from the Inner-City Fund (ICF) International. The DHS uses standard questionnaires and sampling methods to allow comparability of data across countries. The DHS collects comprehensive information on individuals' demographics and health, encompassing areas such as maternal and child health, mortality, nutrition, and the social determinants of health.^17^

### Ethics statement

This study is a secondary analysis of publicly available anonymised multicounty DHS data. Ethical clearance was obtained for all DHS from the respective countries. Informed consent for this study was waived as secondary data were used. This study adhered to the Strengthening the Reporting of Observational Studies in Epidemiology (STROBE) reporting guideline for cross-sectional studies (STROBE Checklist).^18^

### Sampling procedures and sample size

The DHS surveys used a two-stage stratified cluster sampling technique to select the study participants. In the first stage, the first administrative units (e.g., States and Regions) were stratified into urban or rural strata, followed by the selection of Enumeration Areas (EAs) in proportion to the population size of each stratum. In each selected EA, a complete census of households was conducted. In stage two, a fixed number of households were selected using the list of households as a sampling frame.

For this study, the most recent DHS survey data sets from 35 SSA countries were pooled.^19^ The data were collected from eligible women, defined as all females between the ages of 15 and 49 years who were either lived in the households permanently or present on the night before the survey.^19^ In total, we included a weighted sample of 255,891 live births in the five years before each respective survey across 35 SSA countries. Appendix 1 p 1 shows the sample size and response rate for each respective country survey.

### Outcome variable

The main outcome of this study was neonatal mortality, defined as the death of a newborn within the first month of life.^20^ Neonatal mortality was calculated as the number of deaths of newborns among live births within the five years preceding the survey. A death of a newborn within one month was coded as ‘1’ and no death was coded as ‘0’. All neonatal mortality rates in descriptive statistics were presented as per 1000 live births. The focus of our study was driven by the imperative need for targeted interventions to reduce the higher risk of mortality during the first month of life in SSA.

### Modifiable risk factors

The modifiable risk factors were broadly grouped as child, maternal and household factors. Child factors included early initiation of breastfeeding. Maternal factors encompassed maternal education, antenatal care (ANC) visits, place of birth, and maternal tetanus vaccination. Household factors included wealth index, toilet facility, source of drinking water, and type of cooking fuel. The selection of modifiable risk factors was informed by previous studies^21^, ^22^, ^23^ and the availability of relevant data. Table 1 and Appendix 2 pp 2–4 provides the definitions for these modifiable risk factors.

**Table 1:** Definitions of key modifiable risk factors for neonatal mortality among children under five in sub-Saharan Africa.

Modifiable risk factors	Definitions
Child factors	
Early initiation of breastfeeding (EIBF)	EIBF was grouped as ‘1’ = ‘initiated breastfeeding within 1 h of birth’, or ‘2’ = ‘Not initiated breastfeeding within 1 h of birth’
Maternal factors	
Maternal education	Maternal education was grouped as ‘1’ = ‘no or low schooling’, ‘2’ = ‘secondary education or higher’.
Maternal employment	Maternal employment status was grouped as “not working” or “working”
Frequency of antenatal care (ANC) visits	Frequency of ANC visits was grouped as ‘1’ = ‘no or low antenatal care visits, ‘2’ = ‘four and above visits’
Place of birth	Place of birth was grouped as ‘home ‘or ‘health facility birth’
Maternal tetanus toxoid vaccination	Maternal tetanus toxoid vaccination before birth was grouped as ‘1’ = ‘less than 2 doses’, or ‘2’ = ‘two or more doses’.
Household factors	
Household wealth index	Household wealth index was grouped as ‘1’ = ‘poor or medium households’, ‘2’ = ‘rich households ’
Source of drinking water and type of toilet facility	The source of drinking water and type of toilet facility were grouped as ‘1’ = ‘improved’ or ‘2’ = ‘not improved’
Type of cooking fuel	Type of cooking fuel was grouped as ‘1’ = ‘cleaned’ or ‘2’ = ‘not cleaned’

### Potential covariates

We considered potential covariates: the sex of the child (grouped as ‘female’ or ‘male’), the child’s birth order (grouped as ‘first child’, ‘2 to <5 births’, or ‘≥5 births’), maternal age (grouped as ‘15–24 years’, ‘25–34 years’, or ‘35–49 years’), family size (grouped as ‘2–3 members’, ‘4–5 members’, ‘+6 members’) and place of residence (grouped as ‘rural’ or ‘urban’).

### Statistical analysis

The initial analyses involved calculating frequencies and percentages to provide an overview of the study population. In this process, missing data were noted for various variables, including early initiation of breastfeeding (4.6%), maternal education (<0.1%), maternal employment (∼30%), ANC visits (4.4%), place of birth (2.7%), tetanus vaccination (4.0%), family size (0.4%), toilet facilities (∼2%), and cooking fuel (0.2%). Except for maternal employment, which was excluded from the final model, our sensitivity analysis did not reveal any significant impact of missing on our findings. This was followed by fitting the Generalised Linear Latent and Mixed Models (GLLAMM) to determine ORs along with 95% confidence intervals (CIs) for the modifiable risk factors of neonatal mortality. The rationale behind using ORs in this study was based on the rarity assumption (prevalence of less than 10%) observed in cases of neonatal mortality, where ORs and RRs tend to converge.^24^

Our regression analyses followed the principle of VanderWeele's disjunctive cause criterion for confounder selection.^25^^,^^26^ This criterion aims to rectify imbalances in confounder adjustment and causation analysis, specifically addressing overly liberal control of confounders (leading to M-bias) and overly conservative selection of confounders. We grouped the explanatory variables into modifiable risk factors and covariates. Modifiable risk factors were selected based on their significance for outcomes, aligning with the conceptual framework for child survival proposed by Mosley and Chen,^27^ and their amenability to interventions. Covariates, including maternal age, birth order, place of residence, and perceived baby birth size, were selected based on existing literature, exclusion of potential mediators, and their correlation with the outcome, based on previously published studies.^21^^,^^23^

The GLLAMM models involved a three-step process. The initial step involved the development of a null unconditional model, which did not include any predicting variables. In the second step, individual-level factors, such as child, maternal, and household factors, were incorporated into the model. The final model encompassed both individual (child, maternal and household factors) and community-level factors (place of residence). This final model, which included both individual and community-level factors, was selected due to its lower deviance and its superior ability to account for the variation in the outcome variables. Appendix 3 pp 4–7 presents the detailed statistical analysis procedures. For this study, we fitted distinct regression models for singleton and multiple-birth children. This decision was based on the higher mortality risk observed in the latter group and the crucial necessity of identifying specific risk factors for each subgroup.^5^^,^^28^ Additionally, subregional analyses were conducted to examine variations in modifiable risk factors for neonatal mortality across Central, Eastern, Southern, and Western SSA regions in SSA.

Following the identification of modifiable risk factors for neonatal mortality through the application of GLLAMM, we computed PAFs using Miettinen's formula. We selected Miettinen's formula because it is known to yield reliable estimates, even when confounding factors are present, especially when adjusted ORs are applied.^29^ The PAF serves as a metric indicating the proportion of childhood deaths that could potentially be mitigated by addressing the identified modifiable risk factors within the population.^30^ PAF was calculated using the following formula:

PAF=Pc(OR−1)/OR

where Pc is the prevalence of the modifiable risk factor among cases, and OR is the adjusted ORs of neonatal mortality associated with the modifiable risk factors.^29^^,^^30^ Given the modifiable risk factors occur simultaneously within individuals, aggregating the PAF for each specific risk factor may lead to an overestimation of their combined effects.^22^^,^^31^ Based on previously published studies,^32^ we employed communality weights to correct for the overlap of risk factors among participants.^32^

To calculate the communalities, we initially computed the pairwise tetrachoric correlation between all potential modifiable risk factors. Subsequently, a principal components analysis was conducted on the tetrachoric correlation matrix. The communality for each risk factor was determined by the sum of squares of the loadings in all principal components with an eigenvector greater than 1. The weighting of each risk factor was then carried out using the formula: We = 1 − communality. Following this, a combined PAF across the modifiable risk factors was calculated using the specified formula:

$$PAF\;\left(combined\right)=1-\prod_{r=1}^{R}\left(1\;-\;WePAFe\right)$$

where ‘e’ represents each modifiable risk factor, and ‘We’ represents the communality weight of each risk factor. Finally, we estimated the adjusted PAF for each risk factor using the formula:

$$adjusted\;PAFe=\left(PAFe\frac{}{\sum }PAFe\right)\;*\;combined\;PAF\text{.}$$

We weighted the data to avoid imbalances or unequal probabilities in household selections, and non-responses, and to consider clustering and stratification using the ‘svy’ command in STATA (version 15.0, Stata Corp, College Station, TX, USA).^33^ The STATA’s ‘GLLAMM’ package was used to run the regression analysis.^34^ The association between the modifiable risk factors and the outcome variables was presented in terms of ORs along with 95% CIs.

### Role of the funding source

No funding was received for this project.

## Results

**Fig. 1:**
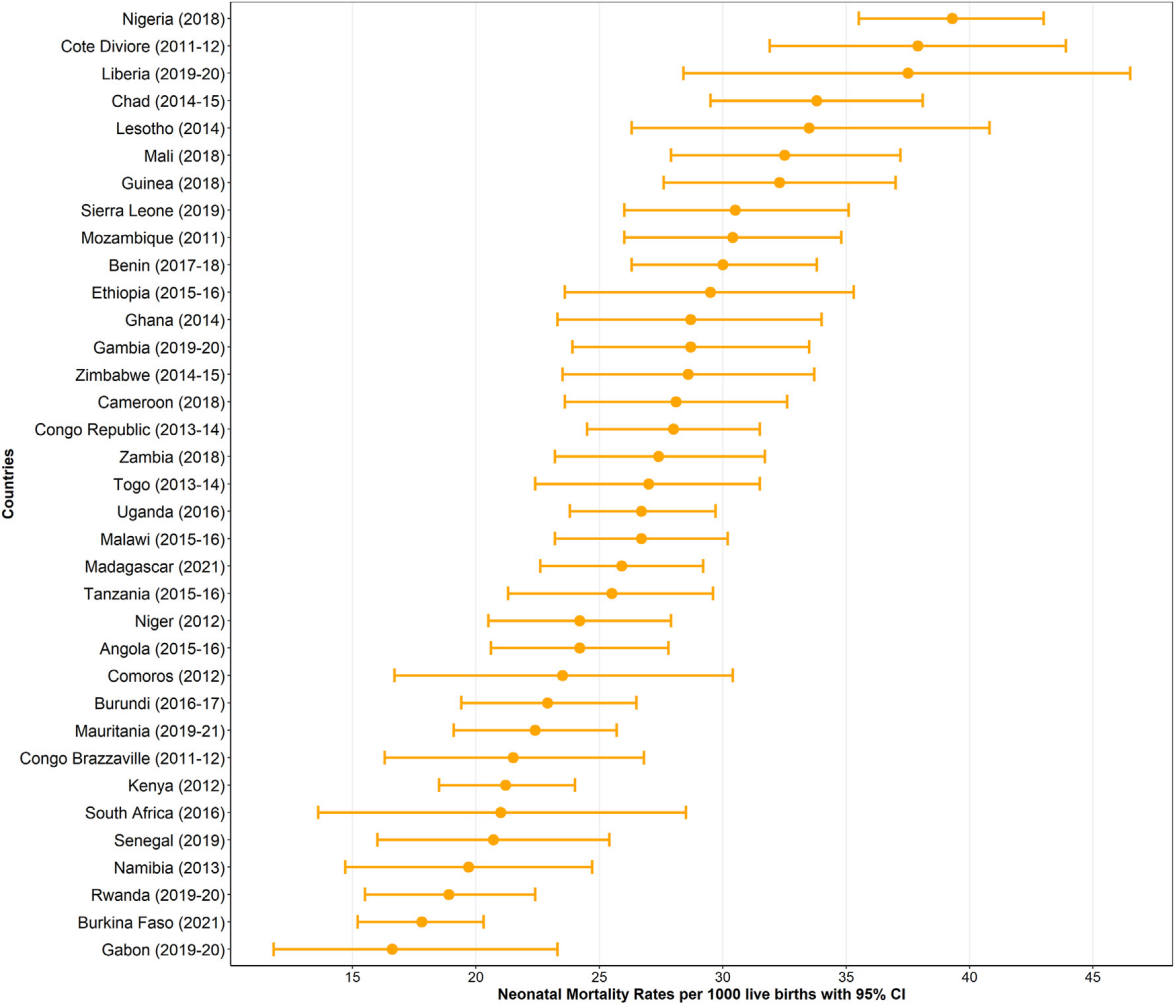
Neonatal mortality rates per 1000 in 35 sub-Saharan Africa countries.

This study included 255,891 live births within five years before the survey with a mean age of 22.9 (±15.7) months. A total of 138,285 (54.0%) mothers started breastfeeding within the first hour of birth, and 174,776 (68.3%) had attained less than primary or no formal education. A total of 177,450 (71.3%) mothers gave birth at a health facility, and 121,093 (49.4%) had two or more tetanus injections before childbirth. A total of 130,454 (51.9%) children resided in households with an unimproved toilet and 167,986 (65.6%) resided in rural households (Table 2). The highest neonatal mortality was observed in Nigeria (neonatal mortality rate [NMR] = 39.3 deaths per 1000 live births, 95% CI: 35.5, 43.0), followed by Côte d'Ivoire (NMR = 37.9 deaths per 1000 live births, 95% CI: 31.9, 43.9), while the lowest NMR was found in Gabon (NMR = 16.6 deaths per 1000 live births, 95% CI: 11.8, 23.3). Fig. 1 and Appendix 4 p 8 show neonatal mortality rates across SSA countries.

**Table 2:** Characteristics of study participants by sex, [N = 255,891].

Variables	Singleton, n (%)	Multiple-births, n (%)	Total, n (%)
Child factors			
Perceived baby birth size			
Below average	39,088 (16.3)	1784 (37.0)	40,872 (16.7)
Average or above average	200,360 (83.7)	3044 (63.)	203,404 (83.3)
Early initiation of breastfeeding			
No	114,820 (45.8)	2786 (54.9)	117,606 (46.0)
Yes	135,997 (54.2)	2288 (45.1)	138,285 (54.0)
Birth order			
One	55,306 (22.1)	–	55,306 (21.6)
2–4 children	120,746 (48.1)	2400 (47.3)	123,146 (48.1)
+5 children	74,765 (29.8)	2673 (52.7)	77,439 (30.3)
Maternal factors			
Maternal age			
15–24 years	75,238 (30.0)	831 (16.4)	76,068 (29.7)
25–34 years	114,458 (45.6)	2618 (51.6)	117,075 (45.8)
35–49 years	61,122 (24.4)	1625 (32.0)	62,747 (24.5)
Maternal education			
No or low education	171,219 (68.3)	3556 (70.1)	174,776 (68.3)
Secondary or higher	79,580 (31.7)	1517 (29.9)	81,098 (31.7)
Maternal employment			
Not working	77,130 (43.5)	1562 (43.2)	78,692 (43.5)
Working	100,142 (56.5)	2057 (56.8)	102,199 (56.5)
Antenatal care			
Three or less visits	102,251 (42.7)	1942 (40.4)	104,193 (42.7)
4 or more visits	137,024 (57.3)	2861 (59.6)	139,885 (57.3)
Place of birth			
Home	70,328 (28.8)	1103 (22.5)	71,431 (28.7)
Health facility	173,648 (71.2)	3801 (77.5)	177,450 (71.3)
Maternal tetanus vaccination			
Less than 2 doses	118,788 (50.6)	2501 (52.0)	123,962 (50.6)
Two or more doses	121,461 (49.4)	2305 (48.0)	121,093 (49.4)
Household factors			
Family size			
2–3 members	35,485 (14.2)	266 (5.3)	35,751 (14.0)
4–5 members	75,859 (30.4)	1210 (23.9)	77,069 (30.2)
+6 members	138,489 (55.4)	3583 (70.8)	142,073 (55.7)
Household wealth			
Poor or medium households	155,782 (62.1)	3161 (62.3)	158,943 (62.1)
Rich households	95,035 (37.9)	1913 (37.7)	96,948 (37.9)
Type of toilet system			
Not improved	127,891 (51.9)	2563 (51.3)	130,454 (51.9)
Improved	118,278 (48.1)	2431 (48.7)	120,709 (48.1)
Source of drinking water			
Not protected	113,441 (45.2)	2274 (44.8)	115,715 (45.2)
Protected	137,376 (54.8)	2800 (955.2)	140,176 (54.8)
Type of cooking fuel			
Not cleaned	210,234 (84.0)	4313 (85.2)	214,547 (16.0)
Cleaned	40,167 (16.0)	748 (14.8)	40,915 (84.0)
Community level factors			
Place of residence			
Urban	86,135 (34.3)	1770 (34.9)	87,905 (34.4)
Rural	164,682 (65.7)	3303 (65.1)	167,986 (65.6)

In singleton children, the strongest risk factor associated with neonatal mortality was delayed initiation of breastfeeding (less than 1 h after birth, OR = 3.68; 95% CI: 3.43, 3.95), followed by mothers lacking two doses of tetanus vaccination during pregnancy (OR = 1.18; 95% CI: 1.11, 1.26), mothers lacking primary education (OR = 1.17; 95% CI: 1.08, 1.26), and infrequent ANC visits (fewer than 3 visits, OR = 1.14; 95% CI: 1.06, 1.21) [Table 3]. Similarly, delayed initiation of breastfeeding was the strongest risk factor associated with neonatal mortality among multiple-birth children (OR = 2.12; 95% CI: 1.76, 2.56), followed by infrequent ANC visits (OR = 1.36; 95% CI: 1.14, 1.63) [Table 4].

**Table 3:** Determinants and population attributable fractions for neonatal deaths among singleton live births in 35 sub-Saharan Africa countries.

Variables	Prevalence of exposure in cases	OR (95% CI)	Unadjusted PAF% (95% CI)	aAdjusted PAF% (95% CI)
Child factors				
Early initiation of breastfeeding				
No	75.4 (73.7, 77.0)	3.68 (3.43, 3.95)	54.91 (52.21, 57.51)	23.88 (15.91, 24.86)
Yes	24.6 (23.0, 26.3)	Ref	Ref	Ref
Maternal factors				
Maternal education				
No or low education	68.7 (66.5, 70.7)	1.17 (1.08, 1.26)	9.98 (4.93, 14.59)	4.34 (1.15, 6.31)
Secondary or higher	31.4 (29.3, 33.5)	Ref	Ref	Ref
Antenatal care				
Three or less visits	46.1 (43.9, 48.3)	1.14 (1.06, 1.21)	5.67 (2.48, 8.38)	2.45 (0.76, 3.63)
4 or more visits	53.9 (51.7, 56.1)	Ref	Ref	Ref
Maternal tetanus vaccination				
Less than 2 doses	53.3 (51.4, 55.2)	1.18 (1.11, 1.26)	8.13 (5.09, 11.39)	3.54 (1.55, 4.92)
Two or more doses	46.7 (44.8, 48.6)	Ref	Ref	Ref
Household factors				
Household wealth				
Poor or medium households	64.1 (61.7, 66.4)	1.02 (0.94, 1.11)	1.26 (−3.94, 6.58)	–
Rich households	35.9 (33.6, 38.3)	Ref	Ref	
Type of toilet system				
Not improved	55.1 (52.9, 57.2)	1.00 (0.93, 1.07)	0.00 (−3.98, 3.74)	–
Improved	44.9 (42.8, 47.2)	Ref	Ref	
Source of drinking water				
Not protected	44.1 (42.1, 46.1)	1.04 (0.97, 1.10)	1.70 (−1.30, 4.19)	–
Protected	55.9 (53.9, 57.9)	Ref	Ref	
Type of cooking fuel				
Not cleaned	83.5 (81.5, 85.3)	1.17 (1.06, 1.31)	12.13 (4.61, 20.19)	5.27 (1.41, 8.73)
Cleaned	16.5 (14.7, 18.5)	Ref	Ref	Ref

PAF: population attributable fraction. OR: odds ratio.

a Adjusted PAF is the relative contribution of each risk factor to the overall PAF when adjusted for communality.

**Table 4:** Determinants and population attributable fractions for neonatal deaths among multiple live births in 35 sub-Saharan Africa countries.

Variables	Prevalence of exposure in cases	OR (95% CI)	Unadjusted PAF% (95% CI)	aAdjusted PAF% (95% CI)
Child factors				
Early initiation of breastfeeding				
No	72.7 (68.0, 76.9)	2.12 (1.76, 2.56)	38.41 (29.36, 46.86)	16.38 (12.11, 20.13)
Yes	27.3 (23.1, 32.1)	Ref	Ref	Ref
Maternal factors				
Maternal education				
No or low education	73.3 (68.7, 77.4)	1.10 (0.89, 1.36)	6.66 (−8.49, 20.49)	–
Secondary or higher	26.7 (22.6, 31.4)	Ref	Ref	
Antenatal care				
Three or less visits	52.5 (47.5, 57.4)	1.36 (1.14, 1.63)	13.90 (5.83, 22.19)	5.93 (2.41, 9.53)
4 or more visits	47.5 (42.6, 52.5)	Ref	Ref	Ref
Maternal tetanus vaccination				
Less than 2 doses	44.6 (39.2, 50.0)	1.09 (0.92, 1.29)	3.68 (−3.4, 11.24)	–
Two or more doses	55.4 (50.0, 60.8)	Ref	Ref	
Household factors				
Household wealth				
Poor or medium households	62.8 (57.3, 68.0)	1.08 (0.87, 1.34)	4.65 (−8.56, 17.25)	–
Rich households	37.2 (32.0, 42.7)	Ref	Ref	
Type of toilet system				
Not improved	53.2 (48.2, 58.1)	0.99 (0.82, 1.19)	−0.54 (−10.58, 9.28)	–
Improved	46.8 (41.9, 51.8)	Ref	Ref	
Type of cooking fuel				
Not cleaned	86.6 (82.8, 89.7)	0.98 (0.75, 1.29)	−1.77 (−27.6, 20.17)	–
Cleaned	13.4 (10.3, 17.2)	Ref	Ref	

PAF: population attributable fraction. OR: odds ratio.

a Adjusted PAF is the relative contribution of each risk factor to the overall PAF when adjusted for communality.

The highest PAFs of neonatal mortality among singleton children (n = 250,817) were attributed to delayed initiation of breastfeeding for more than 1 h of birth (PAF = 23.88%; 95% CI: 15.91, 24.86), uncleaned cooking fuel (PAF = 5.27%; 95% CI: 1.41, 8.73), mother’s lacking formal education (PAF = 4.34%; 95% CI: 1.15, 6.31), less than two maternal tetanus doses before pregnancy (PAF = 3.54%; 95% CI: 1.55, 4.92) and infrequent ANC visits (<3 visits: PAF = 2.45; 95% CI: 0.76, 3.63) (Table 3). The combined PAF showed that these five modifiable risk factors together attributed 39.49% (95% CI: 21.13, 48.44) of neonatal deaths in singleton children in SSA. In multiple-birth children (n = 5074), the largest PAFs of neonatal mortality were attributed to delayed initiation of breastfeeding (PAF = 16.38; 95% CI: 12.11, 20.13) and infrequent ANC visits (<3 visits: PAF = 5.93; 95% CI: 2.41, 9.53) (Table 4). These two risk factors contributed to 22.31% (95% CI: 14.52, 29.66) of neonatal mortality among multiple-birth children in SSA.

Our subregional analyses revealed some variations in modifiable risk factors for neonatal mortality among singleton children. In Central SSA, delayed initiation of breastfeeding (PAF = 32.42; 95% CI: 26.86, 34.37), households with unprotected water supply (PAF = 7.89; 95% CI: 1.96, 9.79), and infrequent ANC visits (PAF = 7.30; 95% CI: 3.92, 10.43) accounted for 47.61% (95% CI: 32.74, 54.59) of neonatal mortality. In Eastern SSA, delayed initiation of breastfeeding (PAF = 24.30; 95% CI: 23.8, 24.49), mothers lacking formal education (PAF = 9.84; 95% CI: 6.96, 12.14), and mothers lacking tetanus vaccination (PAF = 5.92; 95% CI: 4.52, 7.15) contributed to 40.06% (95% CI: 35.28, 43.78) of neonatal mortality (Table 5). Similarly, in Southern SSA, delayed initiation of breastfeeding (PAF = 29.20; 95% CI: 25.59, 29.99), poor households (PAF = 13.25; 95% CI: 8.24, 16.67) and infrequent ANC visits (PAF = 11.87; 95% CI: 9.31, 14.03) were associated with 54.32% (95% CI: 43.14, 60.69) of neonatal mortality. Finally, in Western SSA, delayed initiation of breastfeeding (PAF = 25.96; 95% CI: 23.86, 27.83) and mothers lacking tetanus vaccination (PAF = 3.23; 95% CI: 1.14, 5.30) were linked to 29.19% (95% CI: 25.00, 33.13) of neonatal mortality (Table 6).

**Table 5:** Determinants and population attributable fractions for neonatal deaths among singleton live births in Central and Eastern sub-Saharan Africa countries.

Variables	Central SSA			Eastern SSA		
	Pe (95% CI)	OR (95% CI)	aAdjusted PAF (95% CI)	Pe (95% CI)	OR (95% CI)	aAdjusted PAF (95% CI)
Child factors						
Early initiation of breastfeeding						
No	82.8 (77.6, 87.1)	6.04 (3.18, 11.48)	32.42 (26.86, 34.37)	69.3 (66.1, 72.3)	4.71 (4.03, 5.50)	24.30 (23,8, 24.49)
Yes	17.2 (12.9, 22.5)	Ref	Ref	30.7 (27.7, 33.9)	Ref	Ref
Maternal factors						
Maternal education						
No or low education	52.1 (44.9, 59.3)	1.18 (0.92, 1.50)	–	76.0 (72.8, 79.0)	1.41 (1.25, 1.59)	9.84 (6.96, 12.14)
Secondary or higher	47.9 (40.7, 55.1)	Ref	–	24.0 (21.0, 27.2)	Ref	Ref
Antenatal care						
Three or less visits	39.1 (32.8, 45.7)	1.66 (1.31, 2.12)	7.30 (3.92, 10.43)	54.7 (51.3, 58.0)	1.28 (1.16, 1.41)	5.33 (3.39, 6.98)
4 or more visits	60.9 (54.3, 67.2)	Ref	Ref	45.3 (42.0, 48.7)	Ref	Ref
Maternal tetanus vaccination						
Less than 2 doses	46.4 (39.2, 53.7)	1.39 (1.11, 1.73)	6.11 (1.96, 9.79)	41.6 (38.1, 45.1)	1.47 (1.33, 1.62)	5.92 (4.52, 7.15)
Two or more doses	53.6 (46.3, 60.8)	Ref		58.4 (54.9, 61.9)	Ref	Ref
Household factors						
Household wealth						
Poor or medium households	72.3 (65.8, 78.0)	0.99 (0.75, 1.32)	–	59.1 (55.2, 62.9)	0.83 (0.74, 0.92)	–
Rich households	27.7 (22.0, 34.2)	Ref	–	40.9 (37.1, 44.8)	Ref	–
Type of toilet system						
Not improved	58.7 (51.9, 65.2)	1.07 (0.85, 1.35)	–	58.5 (54.8, 62.1)	1.28 (1.14, 1.42)	5.70 (3.22, 7.60)
Improved	41.3 (34.8, 48.1)		–	41.5 (37.9, 45.2)	Ref	Ref
Source of drinking water						
Not protected	52.6 (45.7, 59.4)	1.47 (1.17, 1.85)	7.89 (1.96, 9.79)	48.9 (45.3, 52.5)	1.15 (1.05, 1.27)	2.84 (1.03, 4.62)
Protected	47.4 (40.6, 54.3)	Ref	Ref	51.1 (47.5, 54.7)	Ref	Ref
Type of cooking fuel						
Not cleaned	65.7 (58.6, 72.2)	0.76 (0.60, 0.97)	–	90.9 (88.0, 93.2)	0.92 (0.78, 1.10)	–
Cleaned	34.3 (27.8, 41.4)	Ref	–	9.1 (6.8, 12.0)	Ref	–

PAF: population attributable fraction. OR: odds ratio.

a Adjusted PAF is the relative contribution of each risk factor to the overall PAF when adjusted for communality.

**Table 6:** Determinants and population attributable fractions for neonatal deaths among singleton live births in Southern and Western sub-Saharan Africa countries.

Variables	Southern SSA			Western SSA		
	Pe (95% CI)	OR (95% CI)	aAdjusted PAF (95% CI)	Pe (95% CI)	OR (95% CI)	aAdjusted PAF (95% CI)
Child factors						
Early initiation of breastfeeding						
No	77.0 (69.5, 83.1)	7.80 (4.13, 14.73)	29.20 (25.59, 29.99)	76.9 (74.6, 79.1)	3.08 (2.66, 3.57)	25.96 (23.86, 27.83)
Yes	23.0 (16.9, 30.5)	Ref	Ref	23.1 (20.9, 25.4)	Ref	Ref
Maternal factors						
Maternal education						
No or low education	34.0 (27.3, 41.39)	1.30 (1.00, 1.69)	–	76.1 (73.5, 78.6)	1.09 (0.98, 1.21)	–
Secondary or higher	66.0 (58.6, 72.7)	Ref	–	23.9 (21.4, 26.5)	Ref	–
Antenatal care						
Three or less visits	38.9 (31.3, 47.1)	3.35 (2.58, 4.34)	11.87 (9.31, 14.03)	43.8 (40.9, 46.8)	1.01 (0.92, 1.11)	–
4 or more visits	61.1 (52.9, 68.7)	Ref	Ref	56.2 (53.2, 59.1)	Ref	–
Maternal tetanus vaccination						
Less than 2 doses	34.7 (27.9, 42.3)	1.94 (1.35, 2.77)	7.31 (3.51, 10.46)	49.6 (46.7, 52.5)	1.15 (1.05, 1.26)	3.23 (1.14, 5.30)
Two or more doses	65.3 (57.7, 72.1)	Ref	Ref	50.5 (47.6, 53.3)	Ref	Ref
Household factors						
Household wealth						
Poor or medium households	67.6 (59.4, 74.7)	1.82 (1.40, 2.36)	13.25 (8.24, 16.67)	63.9 (60.8, 66.9)	1.06 (0.96, 1.17)	–
Rich households	32.5 (25.3, 40.6)	Ref	Ref	36.1 (33.1, 39.2)	Ref	–
Type of toilet system						
Not improved	47.3 (39.4, 55.4)	1.08 (0.86, 1.35)	–	53.7 (50.7, 56.6)	0.91 (0.83, 1.01)	–
Improved	52.7 (44.7, 60.6)	Ref	–	46.4 (43.4, 49.4)	Ref	–
Source of drinking water						
Not protected	31.7 (25.0, 39.3)	1.36 (1.09, 1.69)	3.65 (1.00, 6.21)	41.8 (39.0, 44.6)	1.10 (1.00, 1.20)	–
Protected	68.3 (60.7, 75.0)	Ref	Ref	58.2 (55.4, 61.0)	Ref	–
Type of cooking fuel						
Not cleaned	54.9 (46.6, 62.9)	0.68 (0.52, 0.89)	–	89.1 (86.8, 91.0)	1.12 (0.98, 1.29)	–
Cleaned	45.1 (37.1, 53.4)	Ref	–	10.9 (9.0, 13.2)	Ref	–

PAF: population attributable fraction. OR: odds ratio.

a Adjusted PAF is the relative contribution of each risk factor to the overall PAF when adjusted for communality.

## Discussion

To the best of our knowledge, this was the first study to investigate PAFs of key modifiable risk factors for neonatal mortality in SSA countries. The highest PAFs of neonatal mortality among singleton children were attributed to delayed initiation of breastfeeding (>1 h after birth), use of unclean cooking fuel, mothers lacking formal education, mothers without two doses of tetanus vaccines, and infrequent ANC visits. These five modifiable risk factors accounted for 39.5% of neonatal mortality among singleton children in SSA. Similarly, delayed initiation of breastfeeding and infrequent ANC visits contributed to 22.3% of neonatal mortality among multiple-birth children in SSA. Our analysis at the subregional level revealed that various factors were associated with neonatal mortality across Central, Eastern, Southern and Western regions of SSA.

Previous studies on neonatal mortality primarily relied on relative measures such as ORs or RRs.^12^, ^13^, ^14^ In contrast, this study estimated PAFs, offering direct estimates for effective public health planning and resource allocation.

The World Health Organization and the United Nations Children’s Fund (WHO/UNICEF) recommend early initiation of breastfeeding within the first hour of birth, exclusive breastfeeding for the first 6 months of life, and continued breastfeeding until the child 2 years of age given the nutritional, immunological, economic, and survival benefits of breastfeeding for both mother and child.^35^^,^^36^ Our findings support these recommendations, highlighting that delayed initiation of breastfeeding consistently contributes to the highest PAFs of neonatal mortality across SSA and within four regions: Central, Eastern, Southern, and Western SSA. Previous studies suggested that, in addition to improving early initiation of breastfeeding practices, improving children's survival also requires ensuring that women and children have access to quality healthcare services across the entire spectrum of care, spanning from preconception and pregnancy to delivery, the postnatal period, infancy, and early childhood. This study also underscores the importance of mother’s use of ANC and tetanus vaccination in improving newborn survival, consistent with findings from previous studies in SSA.^6^^,^^7^

Furthermore, our study identifies the significant impact of unclean cooking fuel, inadequate toilet facilities, and the lack of access to clean drinking water on newborn survival. Previous studies suggested that healthy household environments, including proper ventilation, sanitation facilities, and access to clean water sources, play a critical role in shaping child health outcomes, particularly child survival.^37^^,^^38^ For instance, indoor exposure to pollutants from solid fuel combustion, such as ambient particulate matter, poses a direct threat to respiratory health, exacerbating airway diseases and compromising pulmonary defense mechanisms among newborns.^39^ Moreover, newborns, with their higher oxygen consumption and narrower airways, face increased susceptibility to indoor pollutants, leading to elevated risks of acute respiratory tract infections and diminished survival rates. Additionally, an unhealthy household environment is often linked to poverty and poorer living conditions suggesting that neonatal mortality is an outcome of persistent socioeconomic disparities within the SSA regions and countries.

Global progress in reducing under-five mortality has been significant, but neonatal mortality has not decreased proportionally. In 1990, neonatal mortality accounted for 40% of under-five deaths, which then increased to 47% in 2019.^20^ Achieving the SDG health targets by 2030 presents challenges, requiring around $371 billion, excluding costs related to the COVID-19 pandemic and the ongoing global conflicts.^40^ Strategic investments in effective interventions are imperative to prevent neonatal deaths. Our findings underscore the importance of allocating resources towards breastfeeding, tetanus vaccination, addressing household air pollution, enhancing maternal education, and improving perinatal health services. These findings can guide resource allocation, shape public health strategies, and inform policy priorities aimed at reducing neonatal mortality in SSA.

Improving neonatal survival in SSA requires the implementation of a comprehensive continuum of care – spanning the prenatal, intrapartum, and postnatal periods.^41^ This begins with ensuring pregnant mothers receive proper prenatal care, nutritional support and risk stratification, including essential elements such as tetanus vaccinations and micronutrient supplementation. To further enhance neonatal survival, it is imperative to establish well-equipped birthing facilities that offer high-quality care. It is also crucial to prioritise the presence of skilled newborn attendants to ensure safe delivery and timely initiation of breastfeeding. Initiating breastfeeding within the first hour of birth protects the newborn from infection, ensures proper thermal care, positively influences exclusive breastfeeding duration, and stimulates the production of vital colostrum—a crucial source of nutrition and immune protection.^35^ Substantial investments in training and health infrastructure are also imperative for lifesaving interventions, including caesarean delivery and neonatal resuscitation.

To further reduce neonatal mortality, a crucial step is strengthening home-based care during the postnatal period. Female community health workers, residing in the communities they serve, play a key role in providing various postnatal care services and interventions for lactating women and their newborns.^42^ Specifically, these workers are effective in providing culturally appropriate breastfeeding support for the first six months, delivering health information, and fostering a supportive household environment for optimal newborn growth and maternal psychosocial well-being.^42^ To realize this potential, female community health workers need to possess the necessary skills and competencies, and therefore, there is a need for investment in their recruitment, training and deployment.

Calculating PAFs for neonatal mortality rates offers an opportunity to inform resource allocation for reducing newborn and under-five deaths in countries with the highest burden of disease in SSA. The use of nationally representative DHS data sets and the standardised data collection methods and tools has enhanced the generalisability of our findings to the region. The modifiable risk factors examined in this study have also wider policy implications for child health.

Despite the above strengths, this study has limitations. Firstly, PAF estimates assume a causal relationship between each modifiable risk factor and the outcome. In our study, we calculated PAF using the OR for each modifiable risk factor from the cross-sectional data. This approach presented challenges in establishing a temporal relationship between putative modifiable risk factors and outcomes. The application of the Disjunctive Cause Criteria attempts to address the limitations inherent in the cross-sectional nature of the data, but it is primarily theoretical. Nevertheless, our PAF estimates are a parsimonious way of quantifying risk that can complement alternative methods, such as estimating the strength of associations using OR/RRs, in the identification of modifiable risk factors for policy intervention and resource prioritisation. We also recommend future longitudinal studies to confirm the causal pathways linking population-modifiable risk factors to childhood mortality, thereby enhancing our understanding of preventive strategies and interventions.

Secondly, results for specific modifiable risk factors, particularly early initiation of breastfeeding, may have been influenced by recall bias. However, we minimised this bias by using the youngest child within the household. Thirdly, the presence of unmeasured confounders, such as acute respiratory tract infections (ARI) and diarrhoea, might persist, potentially leading to either an over- or underestimation of the PAF. In DHS, ARI and diarrhoea data were based on mothers recalling symptoms occurring in the two weeks preceding the survey. It was challenging to incorporate these variables into this analysis given that all neonatal deaths occurred before the two weeks of the survey. Additionally, the DHS did not collect information on sociocultural and structural risk factors for newborn survival, further limiting our analysis.

Fourth, PAF estimates are based on certain assumptions, including the independence of modifiable risk factors and consistent associations over time.^15^ Yet, these assumptions may be unrealistic due to the complex interplay of socio-economic, cultural, healthcare, maternal, and child-related factors linked to childhood mortality. Nevertheless, PAFs provide a simple and intuitive metric that can complement other methods in identifying modifiable risk factors suitable for policy intervention when prioritizing interventions.

In conclusion, our study identified five modifiable risk factors that would prevent 40% of neonatal mortality in SSA, with some variation across different subregions. Given the current global economic climate, we recommend that policymakers focus on these factors when formulating child health interventions to accelerate the reduction of neonatal mortality. Commencing in countries with the highest burden of neonatal mortality should be a priority. It is imperative to implement a comprehensive continuum of care across the prenatal, intrapartum, and postnatal periods, while also ensuring the active involvement of female community healthcare workers in the continuum of care. This becomes especially crucial in rural Africa, where they will play a vital role in reaching female heads of households.

## Contributors

K.Y.A: conceptualization, data curation, formal analysis, methodology, software, and writing – original draft. S.T., T.A.H, T.K.T, A.F.D, D.B.O, H.M.B, D.M.S, S.A.B, G.D.K, D.B.K, Z.Y.K, E.A, M.G.B, A.S, Y.M, K.T.K, M.M.H, S.M, A.E.A, and A.G.R: writing – review and editing. G.D.K: visualization. A.G.R: supervision. S.T and A.F.D: accessed and verified the data. All authors approved the final submission of the study.

## Data sharing statement

All DHS data are available at https://dhsprogram.com/data/available-datasets.cfm. The DHS provides open access to survey data files for legitimate academic research purposes. To initiate the download process, registration is mandatory. Researchers are required to provide their contact information, research title, and a brief description of the proposed analysis. Approval for dataset access is typically confirmed via email. It is important to note that these datasets are third-party resources and not under the ownership or collection of the authors, who possess no special access privileges. Analysis files created from this data can be requested from the corresponding author.

## Declaration of interests

All authors declare no competing interests.
