# Supplementary material for: Population modifiable risk factors associated with neonatal mortality in 35 sub-Saharan Africa countries: analysis of data from demographic and health surveys
Source: eClinicalMedicine. 2024 Jun 20;73:102682. doi: 10.1016/j.eclinm.2024.102682 (PMC11245992; doi:10.1016/j.eclinm.2024.102682)
Supplement: Appendix 1–4 [file mmc1.docx]

**Appendix 1**

**Table 1: List of countries and sample size included**

| **List of countries** | **Weighted sample** | **Response rates for women’s questionnaire** |
| --- | --- | --- |
| Central SSA |  |  |
| Angola | 8274 | 94.2% |
| Congo (Brazzaville) | 5729 | 98.0% |
| Congo Democratic Republic | 10596 | 98.6% |
| Gabon | 4255 | 95.9% |
| Eastern SSA |  |  |
| Burundi | 8763 | 97.6% |
| Comoros | 1821 | 93.2% |
| Ethiopia | 7343 | 94.6% |
| Kenya | 13292 | 96.6% |
| Madagascar | 9072 | 94.9% |
| Malawi | 13103 | 97.7% |
| Mozambique | 7415 | 99.8% |
| Rwanda | 6167 | 99.7% |
| Tanzania | 6891 | 97.3% |
| Uganda | 9915 | 97.0% |
| Zambia | 7145 | 96.4% |
| Southern SSA |  |  |
| Lesotho | 2513 | 97.1% |
| Namibia | 3756 | 92.3% |
| South Africa | 2968 | 86.2% |
| Zimbabwe | 4841 | 96.2% |
| Western SSA |  |  |
| Benin | 8724 | 97.6% |
| Burkina Faso | 8972 | 98.3% |
| Cameroon | 6407 | 98.0% |
| Chad | 10704 | 92.1% |
| Côte d'Ivoire | 5039 | 98.1% |
| The Gambia | 5234 | 95.1% |
| Ghana | 4022 | 97.3% |
| Guinea | 5278 | 99.0% |
| Liberia | 3922 | 96.4% |
| Mali | 6425 | 97.6% |
| Mauritania | 7475 | 96.2% |
| Niger | 7650 | 95.4% |
| Nigeria | 21370 | 99.3% |
| Senegal | 3958 | 96.1% |
| Sierra Leone | 7087 | 96.7% |
| Togo | 4692 | 97.8% |

**Appendix 2**

**Variables measurement**

***Modifiable risk factors***

The modifiable risk factors were broadly categorized into three groups: child factors (perceived baby birth size and early initiation of breastfeeding), maternal factors (maternal education, maternal employment, antenatal care (ANC) visits, maternal tetanus toxoid vaccination, and place of birth) and household factors (household wealth index, type of toilet system, source of drinking water, and type of cooking fuel). This classification is based on previously published studies in SSA^1-4^.

***Perceived birth size***

The size of the child at birth is classified as small or very small, and average or larger, and is based on the mother’s report of the relative size of the child at birth. For this study, we grouped small or very small as “*below average*” and average or larger as “*average and above birth size*”, based on previously published study^5^.

***Early initiation of breastfeeding***

The initiation of breastfeeding indicators was reported for all children born in the 5 years before the survey. We calculated early initiation of breastfeeding (EIBF) as a percentage of children who started breastfeeding within one hour of birth. For this study, EIBF was grouped as ‘1’ = ‘*initiated breastfeeding within 1 h of birth*’, or ‘2’ = ‘*Not initiated breastfeeding within 1 h of birth*’, based on the previously published studies^5,6^.

***Maternal education***

In DHS, maternal education is generally reported as the highest level of education attended (not necessarily completed) (in categories of no education, primary, secondary, higher than secondary. For this study, we regrouped maternal education as ‘1’ = ‘*no or low schooling*’ or ‘3’ = ‘*secondary education or higher*’.

***ANC visits and place of birth***

The number of antenatal visits is grouped into categories of ‘no or low antenatal care visits’ and 4+ visits, and place of birth was grouped as *home* or *health facility*.

***Wealth Index***

The wealth index represents a combined measurement of a household's overall living standards. It is determined by assessing various factors, including the household's ownership of specific assets like televisions and cars, dwelling features such as flooring material, drinking water source, and toilet facilities. Each asset's importance is calculated using factor scores derived from principal components analysis (PCA). These scores are standardized to have a mean of zero and a standard deviation of one. Based on whether a household owns a particular asset, standardized scores are assigned and summed up. Individuals are then ranked according to their household's total score. Detailed procedures for wealth index construction are available elsewhere^7^.

The population is divided into five equal groups, or quintiles, to establish wealth categories: Lowest, Second, Middle, Fourth, and Highest. For this study, the household wealth index was regrouped as ‘1’ = ‘poor or medium households’, ‘2’ = ‘rich households (fourth and highest quintile)’, based on previously published studies^5,6,8-10^.

***Type of cooking fuel***

The study also considered the type of cooking fuel among modifiable risk factors. For this study, households that used electricity, natural gas, biogas, or kerosene as a cooking fuel were classified as ‘*clean’*, while those households that used charcoal, firewood, or dung were grouped as ‘*not clean’*. This classification is based on previously published studies^11,12^.

***Source of drinking water***

The source of drinking water and type of toilet facility w ere classified as ‘improved’ or ‘not improved’, based on the taxonomy of the WHO and UNICEF Joint Monitoring Programme (JMP) for Water and Sanitation^13^ as applied in past studies^4,6^. Households that used piped water, public tap or standpipe, a tube well or borehole, a protected well/spring, rainwater and/or bottled water were classified as ‘*improved’*. Households that used unprotected well/spring, tanker truck/cart, surface water, and/or sachet water were grouped as ‘*not improved*’^13^.

***Type of toilet system***

The type of toilet facility was also grouped as ‘*improved’* (included flush/pour-flush toilets or flush/pour-flush toilets piped to the sewer system, septic tank or pit latrine; ventilated improved pit (VIP) latrine; pit latrine with slab and/or composting toilet). ‘*Not improved*’ type of facility included flush/pour-flush not piped to sewer, septic tank or pit latrine; pit latrine without slab/open pit; bucket or hanging toilet/hanging latrine and no facility/bush/field^13^.

**Appendix 3**

**Statistical analysis**

***Step 1: descriptive statistics***

Frequencies and percentages were calculated to provide an overview of the study population and the prevalence of neonatal mortality across the study factors. All descriptive analyses accounted for the sampling weights, clustering, and stratification using the 'svy' command in STATA.

***Step 2: Generalised linear latent and mixed models***

***Variable selection***

The modifiable factors were selected based on past literature^1-4^ their importance for the outcomes, availability of data, and the amenability for policy interventions in improving child health and survival. In this study, maternal BMI was excluded due to the missing maternal BMI for some countries (e.g., Angola).

Our selection of covariates was based on: 1) previously published studies^3,4^, (ii) by excluding potential mediators (variables with a potential causal link between modifiable risk factors and outcomes), and (iii) their statistical significance with the outcome. In our analysis, we initially considered the gender of the baby, birth order, maternal age, family size and place of residence as potential covariates, as they were less likely to be part of the causal pathway. However, in the final model, only place of residence and family size retained the significant associations with the outcome.

The Generalised Linear Latent and Mixed Models (GLLAMM) were used to determine the odds ratios (ORs) and 95% confidence intervals for modifiable risk factors of neonatal mortality. Our GLLAMM models were structured in two levels, individual (e.g., child, maternal and household factors) and community levels (place of residence) to account for the hierarchical nature of the data, wherein children under five years old are nested within geographic clusters. The random effects and model fitness outputs are presented in the below table.

Multilevel modelling offers distinct advantages compared to classical single-level logistic regression models. Firstly, it acknowledges the hierarchical nature of data, recognizing that children under-five (level I) is nested within clusters (level II). Failure to account for these hierarchies results in underestimated standard errors of regression coefficients, leading to an overstatement of statistical significance. Secondly, multilevel modelling addresses the dependence of observations within the same clusters; children within the same cluster tend to be more similar than those in different clusters. Lastly, it allows for the simultaneous estimation of cluster-level effects (random effects) and the assessment of associations for community-level predictors, such as place of residence.

The multilevel models were constructed in three steps. Initially, a null unconditional model was developed in stage one, without any study variable. In stage two, individual-level factors (including child, maternal, and household factors) were incorporated into the model. Stage three introduced community-level factors and presented in the results, encompassed both individual and community-level factors. This final model, which included both individual and community-level factors, was chosen due to its minimal deviance and superior ability to explain the variation in the outcome variables.

***Step 3: Population-attributable fractions***

PAF quantifies the percentage of neonatal mortality in SSA that could potentially be averted by addressing the identified modifiable risk factors among the populations^14^. Once the modifiable risk factors for neonatal mortality was identified in the GLLAMM analysis, we calculated the PAFs using Miettinen’s formula. The choice of Miettinen's formula was based on its ability to provide valid estimates even in the presence of confounding, particularly when using adjusted RRs^15,16^.

$$\boldsymbol{PAF = Pc(OR-1)/ OR}$$

Where Pc is the prevalence of the modifiable risk factor among cases, and OR is the adjusted ORs of neonatal mortality associated with the modifiable risk factors. Based on previously published studies^17^, we employed communality weights to correct for the overlap of risk factors among participants^17^.

Initially, we computed the pairwise tetrachoric correlation between all potential modifiable risk factors. Subsequently, a principal components analysis was conducted on the tetrachoric correlation matrix. The communality for each risk factor was determined by the sum of squares of the loadings in all principal components with an eigenvector greater than 1. The weighting of each risk factor was then carried out using the formula: We = 1 − communality.

**Table 2: Commmunality calculations**

| **Variables** | **Comp1** | **Comp2** | **Comp3** | **Square1** | **Square2** | **Square3** | **Communalities (Sum of squares)** | **weight** |
| --- | --- | --- | --- | --- | --- | --- | --- | --- |
| early initiation of breastfeeding | 0.08354 | 0.3008 | 0.2984 | 0.006978932 | 0.09048064 | 0.08904256 | 0.186502132 | 0.813497868 |
| maternal education | 0.06817 | 0.3019 | 0.7258 | 0.004647149 | 0.09114361 | 0.52678564 | 0.622576399 | 0.377423601 |
| antenatal care visits | 0.3977 | -0.09093 | -0.2073 | 0.15816529 | 0.008268265 | 0.04297329 | 0.209406845 | 0.790593155 |
| place of birth | 0.325 | 0.4241 | -0.2599 | 0.105625 | 0.17986081 | 0.06754801 | 0.35303382 | 0.64696618 |
| wealth status | 0.4019 | 0.2459 | 0.07162 | 0.16152361 | 0.06046681 | 0.005129424 | 0.227119844 | 0.772880156 |
| toilet facilities | 0.4022 | -0.3327 | 0.1098 | 0.16176484 | 0.11068929 | 0.01205604 | 0.28451017 | 0.71548983 |
| source of drinking water | 0.3802 | -0.26 | 0.2418 | 0.14455204 | 0.0676 | 0.05846724 | 0.27061928 | 0.72938072 |
| cooking fuel | 0.2301 | -0.276 | 0.31 | 0.05294601 | 0.076176 | 0.0961 | 0.22522201 | 0.77477799 |
| Tetanus vaccination | 0.3594 | -0.2451 | -0.2368 | 0.12916836 | 0.06007401 | 0.05607424 | 0.24531661 | 0.75468339 |

Following this, a combined PAF across the modifiable risk factors was calculated using the specified formula:

$$\boldsymbol{PAF}\left( \boldsymbol{combined} \right)\boldsymbol{=}\boldsymbol{1}\boldsymbol{-}\prod_{\boldsymbol{r=1}}^{\boldsymbol{R}} \boldsymbol{(1}\boldsymbol{-We}\boldsymbol{PAFe)}$$

Where ‘e’ represents each modifiable risk factor, and ‘We’ represents the communality weight of ear risk factor. Finally, we estimated the adjusted PAF for each individual risk factor using the formula:

$\boldsymbol{adjusted PAFe = ([PAFe / \sum PAFe] * combined PAF}$**.**

To address potential imbalances and unequal probabilities in household selections, non-responses, and to account for clustering and stratification, we applied survey weighting to the data using the 'svy' command in STATA (version 15.0, Stata Corp, College Station, TX, USA)^18^. The regression analysis was conducted using the 'GLLAMM' package for STATA^19^. The association between the modifiable risk factors and the outcome variables was presented in terms of ORs along with 95% CIs.

**Appendix 4**

**Table 3: Neonatal mortality rates across 35 SSA countries**

| **Countries** | **Number of neonatal deaths** | **NMR** | **LCI** | **UCI** |
| --- | --- | --- | --- | --- |
| Angola, DHS 2015-16 | 165 | 24.2 | 20.6 | 27.8 |
| Benin, DHS 2017-18 | 200 | 30.0 | 26.3 | 33.8 |
| Burkina Faso, DHS 2021 | 121 | 17.8 | 15.2 | 20.3 |
| Burundi, DHS 2016-17 | 117 | 22.9 | 19.4 | 26.5 |
| Cameroon, DHS 2018 | 131 | 28.1 | 23.6 | 32.6 |
| Chad, DHS 2014-15 | 251 | 33.8 | 29.5 | 38.1 |
| Comoros, DHS 2012 | 44 | 23.5 | 16.7 | 30.4 |
| Congo (Brazzaville), DHS 2011-12 | 86 | 21.5 | 16.3 | 26.8 |
| Congo (Republic), DHS 2013-14 | 226 | 28.0 | 24.5 | 31.5 |
| Cote Diviore, DHS 2011-12 | 121 | 37.9 | 31.9 | 43.9 |
| Ethiopia, DHS 2015-16 | 164 | 29.5 | 23.6 | 35.3 |
| Gabon, DHS 2019-20 | 49 | 16.6 | 11.8 | 23.3 |
| Gambia, DHS 2019-20 | 109 | 28.7 | 23.9 | 33.5 |
| Ghana, DHS 2014 | 59 | 28.7 | 23.3 | 34.0 |
| Guinea, DHS 2018 | 131 | 32.3 | 27.6 | 37.0 |
| Kenya, DHS 2012 | 163 | 21.2 | 18.5 | 24.0 |
| Lesotho, DHS 2014 | 63 | 33.5 | 26.3 | 40.8 |
| Liberia, DHS 2019-20 | 96 | 37.5 | 28.4 | 46.5 |
| Madagascar, DHS 2021 | 174 | 25.9 | 22.6 | 29.2 |
| Malawi, DHS 2015-16 | 237 | 26.7 | 23.2 | 30.2 |
| Mali, DHS 2018 | 151 | 32.5 | 27.9 | 37.2 |
| Mauritania, DHS 2019-21 | 134 | 22.4 | 19.1 | 25.7 |
| Mozambique, DHS 2011 | 188 | 30.4 | 26.0 | 34.8 |
| Namibia, DHS 2013 | 50 | 19.7 | 14.7 | 24.7 |
| Niger, DHS 2012 | 120 | 24.2 | 20.5 | 27.9 |
| Nigeria, DHS 2018 | 608 | 39.3 | 35.5 | 43.0 |
| Rwanda, DHS 2019-20 | 67 | 18.9 | 15.5 | 22.4 |
| Senegal, DHS 2019 | 74 | 20.7 | 16.0 | 25.4 |
| Sierra Leone, DHS 2019 | 177 | 30.5 | 26.0 | 35.1 |
| South Africa, DHS 2016 | 58 | 21.0 | 13.6 | 28.5 |
| Tanzania, DHS 2015-16 | 121 | 25.5 | 21.3 | 29.6 |
| Togo, DHS 2013-14 | 90 | 27.0 | 22.4 | 31.5 |
| Uganda, DHS 2016 | 201 | 26.7 | 23.8 | 29.7 |
| Zambia, DHS 2018 | 117 | 27.4 | 23.2 | 31.7 |
| Zimbabwe, DHS 2014-15 | 88 | 28.6 | 23.5 | 33.7 |

Table 4. Determinants and population attributable fractions for neonatal deaths among singleton live births in Central SSA countries

| **Variables** | **Prevalence of exposure in cases** | **OR (95% CI** | **Unadjusted PAF% (95% CI)** | **Adjusted PAF% (95% CI)** |
| --- | --- | --- | --- | --- |
| Child factors |  |  |  |  |
| Early initiation of breastfeeding |  |  |  |  |
| No | 82.8 (77.6, 87.1) | 6.04 (3.18, 11.48) | 69.09 (53.20, 79.51) | 32.42 (26.86, 34.37) |
| Yes | 17.2 (12.9, 22.5) | Ref | Ref | Ref |
| Maternal factors |  |  |  |  |
| Maternal education |  |  |  |  |
| No or low education | 52.1 (44.9, 59.3) | 1.18 (0.92, 1.50) | 7.93 (-3.91, 19.67) | - |
| Secondary or higher | 47.9 (40.7, 55.1) | Ref | Ref | - |
| Antenatal care |  |  |  |  |
| Three or less visits | 39.1 (32.8, 45.7) | 1.66 (1.31, 2.12) | 15.55 (7.76, 24.14) | 7.30 (3.92, 10.43) |
| 4 or more visits | 60.9 (54.3, 67.2) | Ref | Ref | Ref |
| Maternal tetanus vaccination |  |  |  |  |
| Less than 2 doses | 46.4 (39.2, 53.7) | 1.39 (1.11, 1.73) | 13.02 (3.88, 22.66) | 6.11 (1.96, 9.79) |
| Two or more doses | 53.6 (46.3, 60.8) | Ref | Ref | Ref |
| Household factors |  |  |  |  |
| Household wealth |  |  |  |  |
| Poor or medium households | 72.3 (65.8, 78.0) | 0.99 (0.75, 1.32) | -0.73 (-21.93, 18.91) | - |
| Rich households | 27.7 (22.0, 34.2) | Ref | Ref | - |
| Type of toilet system |  |  |  |  |
| Not improved | 58.7 (51.9, 65.2) | 1.07 (0.85, 1.35) | 3.85 (-9.18, 16.85) | - |
| Improved | 41.3 (34.8, 48.1) |  | Ref | - |
| Source of drinking water |  |  |  |  |
| Not protected | 52.6 (45.7, 59.4) | 1.47 (1.17, 1.85) | 16.82 (6.64, 27.29) | 7.89 (1.96, 9.79) |
| Protected | 47.4 (40.6, 54.3) | Ref | Ref | Ref |
| Type of cooking fuel |  |  |  |  |
| Not cleaned | 65.7 (58.6, 72.2) | 0.76 (0.60, 1.00) | -20.84 (-39.33, 0.00) | - |
| Cleaned | 34.3 (27.8, 41.4) | Ref | Ref | - |

**PAF: population attributable fraction**

**OR: odds ratio**

*** Weighted PAF is the relative contribution of each risk factor to the overall PAF when adjusted for communality**

Table 5. Determinants and population attributable fractions for neonatal deaths among singleton live births in Eastern SSA countries

| **Variables** | **Prevalence of exposure in cases** | **OR (95% CI** | **Unadjusted PAF% (95% CI)** | **Adjusted PAF% (95% CI)** |
| --- | --- | --- | --- | --- |
| Child factors |  |  |  |  |
| Early initiation of breastfeeding |  |  |  |  |
| No | 69.3 (66.1, 72.3) | 4.71 (4.03, 5.50) | 56.67 (53.11, 59.94) | 24.30 (23,8, 24.49) |
| Yes | 30.7 (27.7, 33.9) | Ref | Ref | Ref |
| Maternal factors |  |  |  |  |
| Maternal education |  |  |  |  |
| No or low education | 76.0 (72.8, 79.0) | 1.41 (1.25, 1.59) | 14.20 (5.71, 17.65) | 9.84 (6.96, 12.14) |
| Secondary or higher | 24.0 (21.0, 27.2) | Ref | Ref | Ref |
| Antenatal care |  |  |  |  |
| Three or less visits | 54.7 (51.3, 58.0) | 1.28 (1.16, 1.41) | 9.70 (4.24, 15.19) | 5.33 (3.39, 6.98) |
| 4 or more visits | 45.3 (42.0, 48.7) | Ref |  | Ref |
| Maternal tetanus vaccination |  |  |  |  |
| Less than 2 doses | 41.6 (38.1, 45.1) | 1.47 (1.33, 1.62) | 10.66 (5.51, 15.77) | 5.92 (4.52, 7.15) |
| Two or more doses | 58.4 (54.9, 61.9) | Ref | Ref | Ref |
| Household factors |  |  |  |  |
| Household wealth |  |  |  |  |
| Poor or medium households | 59.1 (55.2, 62.9) | 0.83 (0.74, 0.92) | -12.08 (-19.39, -5.48) | - |
| Rich households | 40.9 (37.1, 44.8) | Ref | Ref | - |
| Type of toilet system |  |  |  |  |
| Not improved | 58.5 (54.8, 62.1) | 1.28 (1.14, 1.42) | 12.80 (6.73, 18.37) | 5.70 (3.22, 7.60) |
| Improved | 41.5 (37.9, 45.2) | Ref | Ref | Ref |
| Source of drinking water |  |  |  |  |
| Not protected | 48.9 (45.3, 52.5) | 1.15 (1.05, 1.27) | 6.80 (1.73, 11.81) | 2.84 (1.03, 4.62) |
| Protected | 51.1 (47.5, 54.7) | Ref | Ref | Ref |
| Type of cooking fuel |  |  |  |  |
| Not cleaned | 90.9 (88.0, 93.2) | 0.92 (0.78, 1.10) | -7.90 (-24.82, 8.47) | - |
| Cleaned | 9.1 (6.8, 12.0) | Ref | Ref | - |

**PAF: population attributable fraction**

**OR: odds ratio**

*** Weighted PAF is the relative contribution of each risk factor to the overall PAF when adjusted for communality**

Table 6. Determinants and population attributable fractions for neonatal deaths among singleton live births in Southern SSA countries

| **Variables** | **Prevalence of exposure in cases** | **OR (95% CI** | **Unadjusted PAF% (95% CI)** | **Adjusted PAF% (95% CI)** |
| --- | --- | --- | --- | --- |
| Child factors |  |  |  |  |
| Early initiation of breastfeeding |  |  |  |  |
| No | 77.0 (69.5, 83.1) | 7.80 (4.13, 14.73) | 67.13 (52.67, 77.46) | 29.20 (25.59, 29.99) |
| Yes | 23.0 (16.9, 30.5) | Ref | Ref | Ref |
| Maternal factors |  |  |  |  |
| Maternal education |  |  |  |  |
| No or low education | 34.0 (27.3, 41.39) | 1.30 (1.00, 1.69) | 7.85 (0.00, 16.90) | - |
| Secondary or higher | 66.0 (58.6, 72.7) | Ref | Ref | - |
| Antenatal care |  |  |  |  |
| Three or less visits | 38.9 (31.3, 47.1) | 3.35 (2.58, 4.34) | 27.29 (19.17, 36.25) | 11.87 (9.31, 14.03) |
| 4 or more visits | 61.1 (52.9, 68.7) | Ref | Ref | Ref |
| Maternal tetanus vaccination |  |  |  |  |
| Less than 2 doses | 34.7 (27.9, 42.3) | 1.94 (1.35, 2.77) | 16.81 (7.23, 27.03) | 7.31 (3.51, 10.46) |
| Two or more doses | 65.3 (57.7, 72.1) | Ref | Ref | Ref |
| Household factors |  |  |  |  |
| Household wealth |  |  |  |  |
| Poor or medium households | 67.6 (59.4, 74.7) | 1.82 (1.40, 2.36) | 30.46 (16.97, 43.05) | 13.25 (8.24, 16.67) |
| Rich households | 32.5 (25.3, 40.6) | Ref | Ref | Ref |
| Type of toilet system |  |  |  |  |
| Not improved | 47.3 (39.4, 55.4) | 1.08 (0.86, 1.35) | 3.50 (-6.41, 14.36) | - |
| Improved | 52.7 (44.7, 60.6) | Ref | Ref | - |
| Source of drinking water |  |  |  |  |
| Not protected | 31.7 (25.0, 39.3) | 1.36 (1.09, 1.69) | 8.39 (2.06, 16.05) | 3.65 (1.00, 6.21) |
| Protected | 68.3 (60.7, 75.0) | Ref | Ref | Ref |
| Type of cooking fuel |  |  |  |  |
| Not cleaned | 54.9 (46.6, 62.9) | 0.68 (0.52, 0.89) | -25.83 (-43.02, -7.77) | - |
| Cleaned | 45.1 (37.1, 53.4) | Ref | Ref | - |

**PAF: population attributable fraction**

**OR: odds ratio**

*** Weighted PAF is the relative contribution of each risk factor to the overall PAF when adjusted for communality**

Table 7. Determinants and population attributable fractions for neonatal deaths among singleton live births in Western SSA countries

| **Variables** | **Prevalence of exposure in cases** | **OR (95% CI** | **Unadjusted PAF% (95% CI)** | **Adjusted PAF% (95% CI)** |
| --- | --- | --- | --- | --- |
| Child factors |  |  |  |  |
| Early initiation of breastfeeding |  |  |  |  |
| No | 76.9 (74.6, 79.1) | 3.08 (2.66, 3.57) | 51.93 (46.55, 56.94) | 25.96 (23.86, 27.83) |
| Yes | 23.1 (20.9, 25.4) | Ref | Ref | Ref |
| Maternal factors |  |  |  |  |
| Maternal education |  |  |  |  |
| No or low education | 76.1 (73.5, 78.6) | 1.09 (0.98, 1.21) | 6.28 (-1.5, 13.64) | - |
| Secondary or higher | 23.9 (21.4, 26.5) | Ref | Ref | - |
| Antenatal care |  |  |  |  |
| Three or less visits | 43.8 (40.9, 46.8) | 1.01 (0.92, 1.11) | 0.43 (-3.56, 4.64) | - |
| 4 or more visits | 56.2 (53.2, 59.1) | Ref | Ref | - |
| Maternal tetanus vaccination |  |  |  |  |
| Less than 2 doses | 49.6 (46.7, 52.5) | 1.15 (1.05, 1.26) | 6.47 (2.22, 10.83) | 3.23 (1.14, 5.30) |
| Two or more doses | 50.5 (47.6, 53.3) | Ref | Ref | Ref |
| Household factors |  |  |  |  |
| Household wealth |  |  |  |  |
| Poor or medium households | 63.9 (60.8, 66.9) | 1.06 (0.96, 1.17) | 3.62 (-2.53, 9.72) | - |
| Rich households | 36.1 (33.1, 39.2) | Ref | Ref | - |
| Type of toilet system |  |  |  |  |
| Not improved | 53.7 (50.7, 56.6) | 0.91 (0.83, 1.01) | -5.31 (-10.38, 0.56) | - |
| Improved | 46.4 (43.4, 49.4) | Ref | Ref | - |
| Source of drinking water |  |  |  |  |
| Not protected | 41.8 (39.0, 44.6) | 1.10 (1.00, 1.20) | 3.80 (0.00, 7.43) | - |
| Protected | 58.2 (55.4, 61.0) | Ref | Ref | - |
| Type of cooking fuel |  |  |  |  |
| Not cleaned | 89.1 (86.8, 91.0) | 1.12 (0.98, 1.29) | 9.55 (-1.77, 20.46) | - |
| Cleaned | 10.9 (9.0, 13.2) | Ref | Ref | - |

**PAF: population attributable fraction**

**OR: odds ratio**

*** Weighted PAF is the relative contribution of each risk factor to the overall PAF when adjusted for communality**

**References**

1. Ahmed KY, Agho KE, Page A, Arora A, Ogbo FA. Mapping Geographical Differences and Examining the Determinants of Childhood Stunting in Ethiopia: A Bayesian Geostatistical Analysis. *Nutrients* 2021; **13**(6).

2. Ahmed KY, Page A, Arora A, Ogbo FA. Associations between infant and young child feeding practices and acute respiratory infection and diarrhoea in Ethiopia: A propensity score matching approach. *PloS one* 2020; **15**(4): e0230978.

3. Ogbo FA, Page A, Idoko J, Agho KE. Population attributable risk of key modifiable risk factors associated with non-exclusive breastfeeding in Nigeria. *BMC public health* 2018; **18**(1): 247.

4. Ahmed KY, Dadi AF, Ogbo FA, et al. Population-Modifiable Risk Factors Associated With Childhood Stunting in Sub-Saharan Africa. *JAMA Network Open* 2023; **6**(10): e2338321-e.

5. Ahmed KY, Agho KE, Page A, et al. Mapping Geographical Differences and Examining the Determinants of Childhood Stunting in Ethiopia: A Bayesian Geostatistical Analysis. *Nutrients* 2021; **13**(6).

6. Ahmed KY, Page A, Arora A, Ogbo FA, Global M, Child Health Research c. Associations between infant and young child feeding practices and acute respiratory infection and diarrhoea in Ethiopia: A propensity score matching approach. *PloS one* 2020; **15**(4): e0230978.

7. The DHS Program. Wealth Index Construction. 2016. <https://www.dhsprogram.com/topics/wealth-index/Wealth-Index-Construction.cfm>.

8. Ahmed KY, Abrha S, Page A, et al. Trends and determinants of underweight and overweight/obesity among urban Ethiopian women from 2000 to 2016. *BMC public health* 2020; **20**(1): 1276.

9. Ahmed KY, Ross AG, Hussien SM, Agho KE, Olusanya BO, Ogbo FA. Mapping Local Variations and the Determinants of Childhood Stunting in Nigeria. *International journal of environmental research and public health*, 2023. (accessed.

10. Ahmed KY, Rwabilimbo AG, Abrha S, et al. Factors associated with underweight, overweight, and obesity in reproductive age Tanzanian women. *PloS one* 2020; **15**(8): e0237720.

11. Naz S, Page A, Agho KE. Household Air Pollution and Under-Five Mortality in Bangladesh (2004-2011). *International journal of environmental research and public health* 2015; **12**(10): 12847-62.

12. Naz S, Page A, Agho KE. Household air pollution from use of cooking fuel and under-five mortality: The role of breastfeeding status and kitchen location in Pakistan. *PloS one* 2017; **12**(3): e0173256-e.

13. Girma M, Hussein A, Norris T, et al. Progress in Water, Sanitation and Hygiene (WASH) coverage and potential contribution to the decline in diarrhea and stunting in Ethiopia. *Maternal & child nutrition* 2021: 1.

14. Miettinen OS. Proportion of disease caused or prevented by a given exposure, trait or intervention. *American journal of epidemiology* 1974; **99**(5): 325-32.

15. Ahmad K, Rasmus Oestergaard N, Mohammad Ali M. Methods matter: population attributable fraction (PAF) in sport and exercise medicine. *British Journal of Sports Medicine* 2020; **54**(17): 1049.

16. Khosravi A, Nazemipour M, Shinozaki T, Mansournia MA. Population attributable fraction in textbooks: Time to revise. *Global Epidemiology* 2021; **3**: 100062.

17. Sue See R, Thompson F, Russell S, et al. Potentially modifiable dementia risk factors in all Australians and within population groups: an analysis using cross-sectional survey data. *The Lancet Public Health* 2023; **8**(9): e717-e25.

18. StataCorp. Stata survey data reference manual release 15. College Station, Texas A Stata Press Publication StataCorp LLC; 2017.

19. Rabe-Hesketh S. GLLAMM: Stata program to fit generalised linear latent and mixed models. 2000
